# The Impact of the Combination of Proteolytic Enzyme and Rutin on the Post-operative Management of Inflammation: A Randomized, Controlled, Double-Blind, Comparative Clinical Trial

**DOI:** 10.7759/cureus.72420

**Published:** 2024-10-26

**Authors:** Piyush C Rathi, Chandrakant L Rathi, Shilpa P Risbud, Gayatri P Ganu

**Affiliations:** 1 Research and Development, Advanced Vital Enzymes Private Limited (ADVENZA), Thane, IND; 2 Pharmacology, Mprex Healthcare Pvt. Ltd., Pune, IND

**Keywords:** inflammation, pain, protease, proteolytic enzyme, rutin

## Abstract

Introduction

Post-operative wound infections are a common complication that may impede recovery and require longer hospital stays. These infections substantially impact patient outcomes, increasing the likelihood of complications and healthcare expenses. Effective management of inflammation and pain is crucial for optimizing post-operative care.

Method

In this study, a total of 65 patients were randomized to either one of the three groups - EnMax tablet (Advanced Vital Enzymes Private Limited (ADVENZA), Thane, India) along with standard of care (SOC), Placebo tablet along with SOC, and Marketed reference preparation along with SOC. Follow-up visits were conducted on days 1, 3, 6, and 8.

Results

On day 8, EnMax demonstrated a statistically significant reduction in inflammation scores of 91.49%, compared to 80% with the marketed reference preparation and 63.64% with placebo. The EnMax group also showed a greater reduction in Visual Analog Scale (VAS) pain scores of 95.29% compared to the marketed reference preparation of 86.42%. Significant reductions in C-reactive protein (CRP) of 51.24% and erythrocyte sedimentation rate (ESR) at 59.80% were observed in the EnMax group, while the marketed reference preparation showed reductions of 35.23% and 46.38%, respectively. Both patient and investigator assessments favored EnMax over the marketed reference and placebo formulation. The study reported no adverse events, with stable vital signs and clinically insignificant changes in complete blood count (CBC), indicating the safety and tolerability of the investigational products.

Conclusion

EnMax, a combination of systemic microbial and plant proteases with the herbal component rutin, achieved significant reductions in post-operative inflammation, pain, and analgesic use, and improved overall patient and investigator assessment scores, outperforming placebo and demonstrating comparable, slightly superior efficacy to the marketed reference preparation. This study not only serves as a valuable guideline for current clinical practice in managing post-surgical inflammation and pain but also establishes a robust baseline for further research in postoperative care and other inflammatory conditions.

## Introduction

Post-operative wound infections represent a significant and pervasive challenge in modern healthcare, often complicating recovery and extending hospital stays. These infections are driven by intricate biological interactions at the molecular level, contributing to high rates of morbidity, mortality, and healthcare costs [1]. Inadequate tissue healing is a widespread medical challenge of surgical wound infections accounting for high morbidity and mortality, as well as a debilitating quality of life. Epidemiological data indicates that surgical site infections (SSI) of wounds account for over two million nosocomial infections in hospitalized patients in the United States [2]. Recent studies reported that the SSI rate ranges from 19.4% to 36.5% all over the world, whereas it ranges from 3% to 12% in India [3]. Contemporary challenges in assessing postoperative wound infections stem from diverse specialties, operations, and shorter hospital stays. Centers for Disease Control and Prevention (CDC's) 2018 data on SSI indicates an extra 11 hospitalization days for affected patients, leading to increased financial burden. SSI rates vary by surgery type: 2.1 per 1000 for clean surgeries, 3.3 per 1000 for clean-contaminated surgeries, 6.4 per 1000 for contaminated surgeries, and 7.1 for dirty surgeries [4,5]. In addition to causing burden through prolonged hospitalizations, loss of mobility, compromising quality of life, requiring support, and sometimes causing nosocomial infections [6].

Proteolytic enzymes, employed since ancient times, aid tissue repair by enhancing the resolution of inflammatory symptoms and expediting wound recovery [7]. These enzymes, including matrix metalloproteases (MMPs) and caspases, act by modifying the extracellular and intracellular environments. MMPs degrade the extracellular matrix, process cytokines, and growth factors, and regulate skin homeostasis by influencing keratinocyte desquamation in the epidermis [8]. Physiological healing processes triggered by injuries, fractures, and burns undergo physiological healing in four phases: hemostasis and coagulation, inflammation, proliferation, and remodeling. Proteolytic enzymes enable plasmin function, which is required for unblocking the microcirculation and relieving edema while also starting the healing process [9].

In post-operative care, managing inflammation and pain is crucial to prevent prolonged hospital stays. Inflammation, a natural protective response, aids in cellular debris disposal for tissue protection and repair. Pain and inflammation primarily result from inflammatory responses. While commonly used, anti-microbial agents, non-steroidal anti-inflammatory drugs (NSAIDs), and steroids pose risks and side effects in inflammation management [10].

In the present study, EnMax (Advanced Vital Enzymes Private Limited (ADVENZA), Thane, India) is a combination of protease, rutin, and other components. These components are thought to synergistically enhance immune function, reduce inflammation by degrading plasma proteins and inflammatory mediators, and promote overall health by boosting metabolism and eliminating toxins.

This study seeks to contribute valuable insights into the clinical application of proteolytic enzymes in postoperative wound healing and pain reduction. By comparing the efficacy of EnMax tablets with a placebo and a marketed preparation as an adjuvant to standard treatment, the research aims to provide evidence-based recommendations for improving patient outcomes in post-surgery care.

## Materials and methods

Study design

This was a randomized, double-blind, parallel-group, controlled, multicentric, comparative clinical study to evaluate the impact of interventional enzymes on post-operative wound healing. The study groups received either one of the following treatments: EnMax tablet along with standard of care (SOC), Placebo tablet of EnMax along with SOC, or Marketed reference preparation (trypsin, bromelain, and rutoside trihydrate tablets) along with SOC. The duration of the treatment period was seven days. Ethical approval for the study was obtained from the Institutional Ethics Committee (IEC) of Lokmanya Medical Research Centre, Chinchwad. The clinical trial was registered with the Clinical Trial Registry-India (CTRI) under the registration number CTRI/2024/03/063636 (Registered on: 05/03/2024). The study was conducted at two sites - Lokmanya Medical Research Centre and Hospital, Pune, and Sangvi Multispeciality Hospital Pvt. Ltd., Pune as per the approved protocol, Declaration of Helsinki, and Good Clinical Practices guidelines. Clinical trial data were collected between 18/04/2024 and 27/05/2024. The compositions of the investigational products are depicted in (Table 1).

**Table 1:** Investigational products composition

Investigational product details		
EnMax	Marketed reference product	Placebo
Microbial & Plant Proteolytic Enzymes, (20,00,000 FCC PU) Rutin (100 mg)	Trypsin (48 mg) Bromelain (90 mg) Rutoside (100 mg)	Microcrystalline cellulose

Inclusion criteria

Patients aged 25 to 50 years (both inclusive) were included in the study. Patients who were electively posted for clean surgeries, such as dental surgery, orthopedic surgery, cosmetic surgery, laparoscopic surgery, etc., were enrolled. Only those patients without any suspected or confirmed infection and not receiving treatment for the same were considered for enrolment. Additionally, patients willing to provide informed consent for the study were included. The details of the trial were explained to all patients, and written consent was obtained before the surgery. Post-surgery efficacy and safety endpoints were assessed.

Exclusion criteria

Patients were excluded if they had any allergy, sensitivity, or contraindication to any interventional product. Patients undergoing emergency, clean-contaminated, contaminated, and dirty surgical procedures were not included. Those with hepatic and/or renal disorders, bleeding disorders, menorrhagia, hematuria, and hematemesis were excluded. Patients with a history of gastric ulcer or bleeding diathesis were not considered. Patients who were currently receiving cytotoxic therapy, or had received it within the last three months, were excluded. Additionally, those treated with any investigational drug in the preceding four weeks were not included. Female patients with a positive pregnancy test or who were lactating were excluded. Patients with uncontrolled diabetes mellitus or any other metabolic disorder were not included. Seriously ill and moribund patients with complications were excluded. Patients who were unable to comply with the treatment regimen or had any other condition that, in the opinion of the investigator, did not justify their inclusion in the study were also excluded. 

Sample size

As per research and clinical judgment of the investigator 65 patients in 2:2:1 (EnMax: Placebo: Marketed reference) were intended to be analyzed at the end of the study.

Methodology

After a written informed consent process on screening visits, the patient's demographic details were recorded. This was a randomized, double-blind, parallel-group, comparative, multicenter, controlled clinical study designed to assess the impact of interventional enzymes on post-operative wound healing.

**Figure 1:**
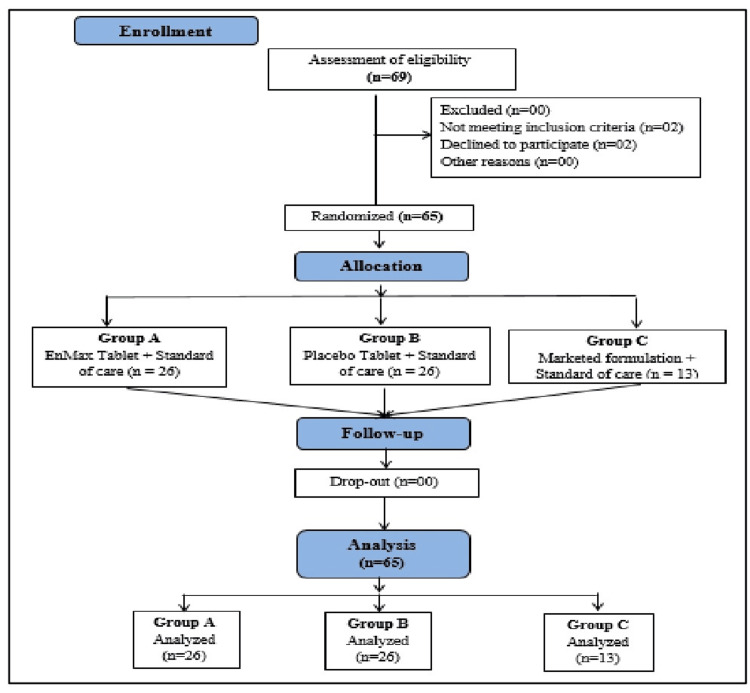
Consolidated Standards of Reporting Trials (CONSORT) diagram for the study.

In this study, a total of 65 patients were randomized to either one of the following groups: Group A (26 patients) receiving the EnMax tablet along with SOC, Group B (26 patients) receiving the Placebo tablet along with SOC, and Group C (13 patients) receiving the Marketed reference preparation along with SOC, in a 2:2:1 ratio, as depicted in (Figure 1). The treatment duration was seven days, and the efficacy of the investigational products was compared between the groups.

Concomitant diseases and medication assessments were conducted during the screening. The evaluation of the verbal rating scale (VRS) inflammation score and VAS pain score was done on days 1, 3, 6, and 8. Inflammatory biomarkers CRP and ESR were evaluated on days 1 and 8. Assessment of changes in analgesics as rescue medications was done on days 1 (visit 1), 3 (visit 2), 6 (visit 3), and 8 (visit 4). Assessment of changes in surgical wound-related symptoms such as erythema, local irritation, discharge, induration, and tenderness based on a 4-point scale was done on days 1, 3, 6, and 8. Patient and physician Global Assessment of Response to Therapy was done at the end of the study (day 8) on a 5-point scale.

Assessment of laboratory parameters such as CBC was done at baseline and end of the study. The safety of the investigational treatment, including adverse events (AEs) and serious adverse events (SAEs), was monitored from baseline to the end of the study. Treatment compliance and tolerability of the investigational products were assessed throughout the study.

Blood samples were collected by a trained phlebotomist at each study site during visit 1 and visit 4. Assessment samples were processed at at centralized National Accreditation Board for Testing and Calibration Laboratories (NABL)-accredited laboratory.

Statistical analysis

Statistical analysis was performed using Statistical Package for Social Sciences (SPSS) version 10.0 (SPSS Inc., Chicago, USA) The data's normality was assessed by using the Kolmogorov-Smirnov test. Demographic data was represented as Mean ± S.D. The primary and secondary endpoints were analyzed by using a Student t-test, Mann-Whitney U Test, Wilcoxon Signed-Ranks Test, and chi-square test. Significance was set at p< 0.05.

## Results

Assessment of demographics 

A total of 69 patients were screened, four were screen failure and 65 were randomized in a 2:2:1 (EnMax: Marketed: Placebo) ratio and all the patients completed the study (Figure 1).

The study included 26 patients in the EnMax group, 13 in the Marketed reference group, and 26 in the Placebo group. The average age and gender distribution for each group are presented in Table 2.

**Table 2:** Demographic details Data is represented as Mean ± S.D.

Demographic Details	EnMax (n=26)	Marketed reference (n=13)	Placebo (n=26)
Male	16	09	18
Age (Average ± SD)	45.438±8.278	34.889±9.171	43±7.985
Female	10	04	08
Age (Average ± SD)	41.8±8.677	45±3.742	33.750±10.634

Assessment of investigator reported inflammation score using verbal rating scale over time

Inflammation was assessed using a VRS, where scores were assigned as follows: 0 for no swelling, 1 for mild swelling confined to the surgery area, 2 for moderate swelling beyond the surgery area, and 3 for severe swelling spreading beyond the surgery area.

The EnMax group showed a significant reduction in inflammation over the course of the study. At visit 2, there was a 55.32% reduction in inflammation score, which further improved to 70.21% by visit 3, and reached 91.49% by visit 4, with all reductions being statistically significant. The Marketed reference group also demonstrated a notable and significant reduction in inflammation score, achieving a 60% reduction at visit 2, 68% at visit 3, and 80% by visit 4. These reductions were significant as well.

**Figure 2:**
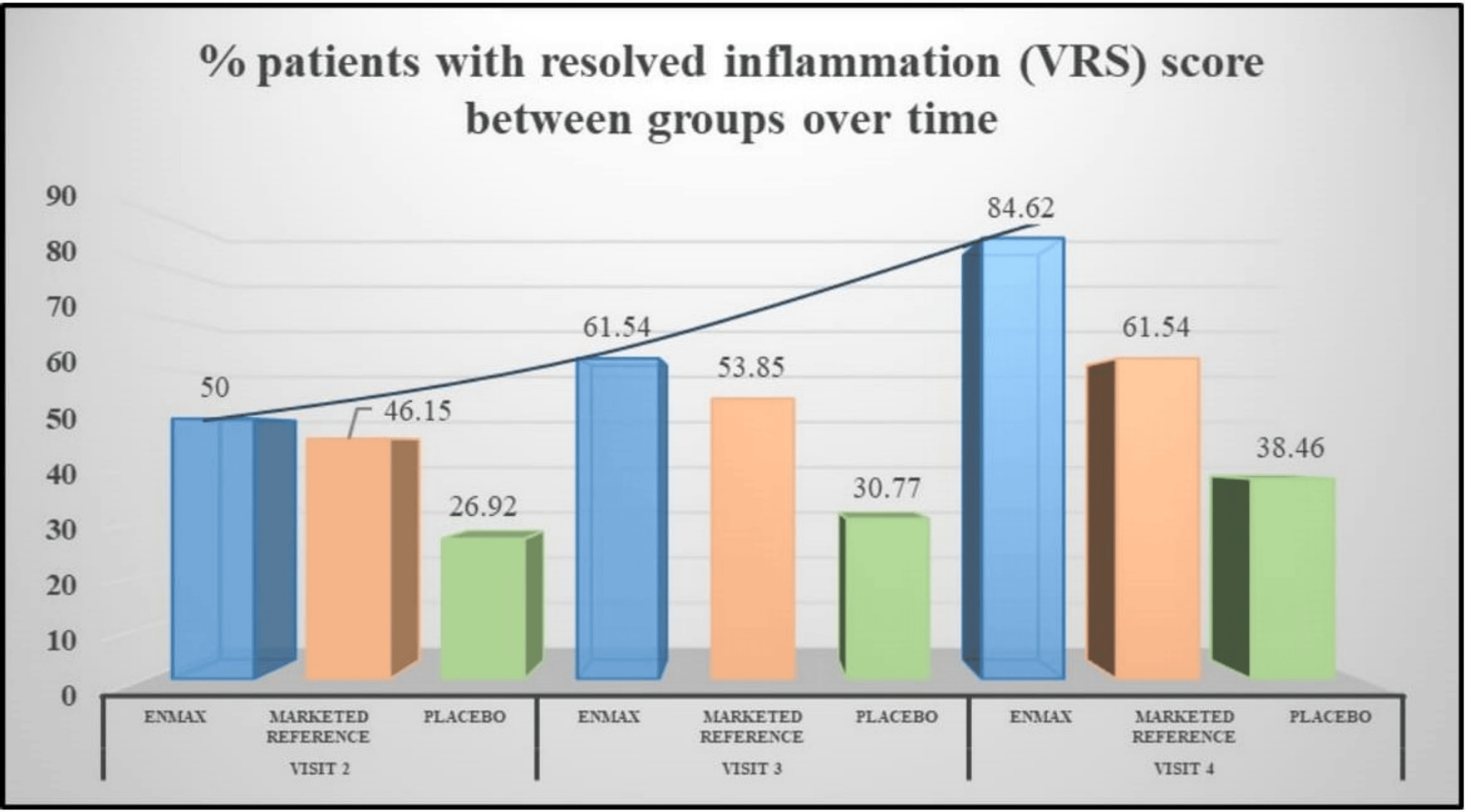
Assessment of changes in the percentage of patients with resolved inflammation between groups VRS: verbal rating scale

The Placebo group exhibited the least reduction in inflammation score, with a 47.73% reduction at visit 2, 52.27% at visit 3, and 63.64% by visit 4. However, these reductions were significant at all visits (Table 3). However, the between-group differences in inflammation score between EnMax vs. Placebo and EnMax vs. Marketed reference did not show any statistically significant difference. Overall, the EnMax group displayed the highest percentage reduction in inflammation score, followed by the Marketed reference group, with the Placebo group showing the least reduction (Figure 2).

**Table 3:** Assessment of inflammation score using verbal rating scale between groups The data was analyzed by between-group, Mann-Whitney U Test, and within the group by the Wilcoxon Signed-Ranked Test. Significant at p< 0.05. * - denotes within group p values <0.001 $ - denotes p values between EnMax v/s marketed reference group # - denotes p values between EnMax v/s placebo group.

Group	Visit 1	Visit 2	Visit 3	Visit 4
EnMax	1.808±0.634	0.808±0.981^*^ (55.32%)	0.538±0.811^*^ (70.21%)	0.154±0.368^*^ (91.49%)
Marketed reference	1.923±0.641	0.769±0.927^*^ (60%)	0.615±0.870^*^ (68%)	0.385±0.506 (80%)
^$^P-value	0.646	0.780	0.928	0.897
Placebo	1.692±0.679	0.885±0.711^*^ (47.73%)	0.808±0.694^*^ (52.27%)	0.615±0.496^*^ (63.64 %)
^#^P-value	0.660	0.352	0.112	0.009

Assessment of visual analog pain scale between groups

**Figure 3:**
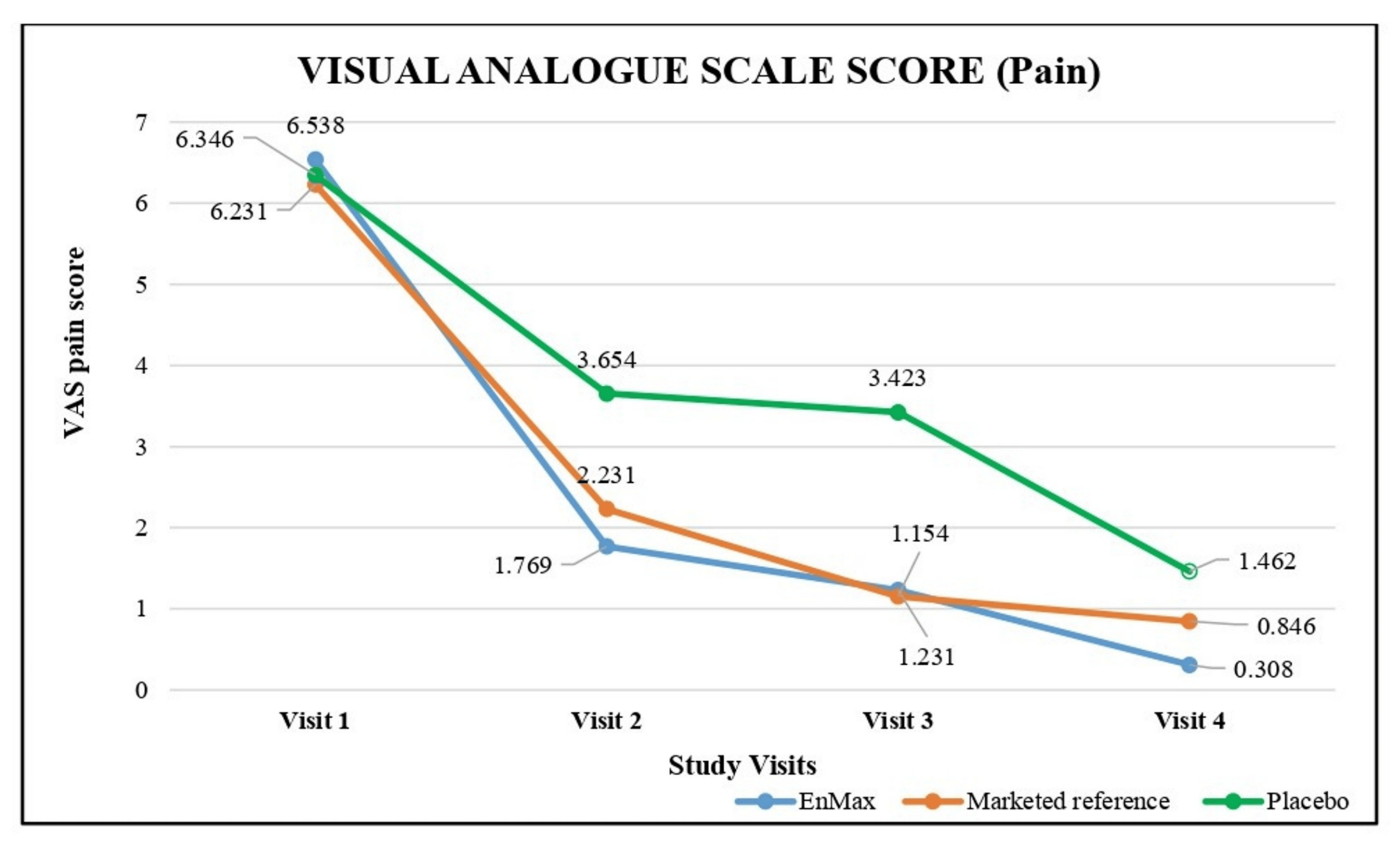
Assessment of visual analog pain scale (VAS) score between groups

The reduction in pain scores across the three study groups (EnMax, Marketed reference, and Placebo) was assessed using the Visual Analog Pain Scale, where scores ranged from 0 (no pain) to 10 (worst pain imaginable) as detailed in (Table 4) and depicted in (Figure 3).

**Table 4:** : Assessment of visual analog pain scale score between groups The data was analyzed by between-group, Mann-Whitney U Test, and within the group by the Wilcoxon Signed-Ranked Test. Significant at p< 0.05. * - denotes within group p values <0.001 $ - denotes p values between EnMax v/s marketed reference group # - denotes p values between EnMax v/s placebo group.

Group	Visit 1	Visit 2	Visit 3	Visit 4
EnMax(n=26)	6.538± 0.508	1.769±1.904^*^ (72.94%)	1.231±1.632^*^ (81.18%)	0.308±0.679^*^ (95.29%)
Marketed reference (n=13)	6.231±0.725	2.231±1.363^*^ (64.20%)	1.154±1.345 (81.48%)	0.846±1.463 (86.42%)
^$^P-value	0.263	0.232	0.542	0.107
Placebo (n=26)	6.346±0.629	3.654±2.097^*^ (42.42%)	3.423±2.230^*^ (46.06 %)	1.462±1.174^*^ (76.97 %)
^#^P-value	0.358	< 0.001	0.001	0.001

The EnMax group showed a significant and consistent reduction in pain score evident from visit 2 over the course of the study. At visit 2, there was a 72.94% reduction in pain score, which further improved to 81.18% by visit 3, and reached 95.29% by visit 4, with all reductions being statistically significant.

The Marketed reference group also demonstrated a substantial and significant reduction in pain score, achieving a 64.20% reduction at visit 2, 81.48% at visit 3, and 86.42% by visit 4. These reductions were significant as well.

The Placebo group exhibited the least yet significant reduction in pain, with a 42.42% reduction at visit 2, 46.06% at visit 3, and 76.97% by visit 4. The reductions at all visits were significant.

Overall, the EnMax group displayed the highest percentage reduction in pain, followed by the Marketed reference group, with the Placebo group showing the least reduction. The between-group differences between EnMax vs. Marketed reference did not show any significant difference and EnMax vs. Placebo showed significant difference.

Assessment of changes in inflammatory biomarkers between groups

The study assessed changes in inflammatory biomarkers, specifically CRP and ESR levels, across the EnMax, Placebo, and Marketed reference groups over four visits. The results are detailed in Table 5.

**Table 5:** Assessment of changes in inflammatory biomarker levels between groups Values represent the mean score ± SD. The data was analyzed using the independent Student's t-test for between-group comparisons and the dependent t-test for within-group comparisons. Significant at p< 0.05. * - denotes within group p values <0.001

Visits	EnMax	Placebo	Marketed reference
C-reactive protein levels (mg/dL)			
Visit 1	10.84± 2.95	10.60±3.26	11.02±3.79
Visit 4	5.28±2.08 (51.24%)	8.18±2.60^*^ (22.82 %)	7.14±2.09 (35.23 %)
P-value	<0.001	<0.001	<0.001
Visit 1	22.77± 5.41	21.58±5.29	23.38 ±5.88
Visit 4	9.15±4.79 (59.80 %)	15.19±6.32^*^ (29.59%)	12.54±5.06 (46.38 %)
P-value	<0.001	<0.001	<0.001

CRP Levels

The EnMax group demonstrated a significant 51.24% reduction in CRP levels from visit 1 to visit 4. In comparison, the Placebo group showed a 22.82% decrease, and the Marketed reference group exhibited a 35.23% reduction. All groups experienced significant reductions in CRP levels compared to visit 1, with the EnMax group showing the highest and most significant reduction as revealed by between-group analysis.

ESR Levels

Similarly, the EnMax group showed a substantial 59.80% reduction in ESR levels from visit 1 to visit 4. The Placebo group had a 29.59% decrease, while the Marketed reference group saw a 46.38% reduction. Again, all groups experienced significant reductions in CRP levels compared to visit 1, with the EnMax group showing the highest and most significant reduction as revealed by between-group analysis.

These findings indicate that the EnMax group experienced the most significant decreases in both CRP and ESR levels, followed by the Marketed reference group, while the Placebo group showed the least reduction in these inflammatory biomarkers over the course of the study.

Assessment of use of analgesics as rescue medication between groups

The use of analgesics as rescue medication was assessed across the EnMax, Marketed reference, and Placebo groups over four visits. For patients from the EnMax group, at visit 1, 100% of patients required analgesic rescue medication. This decreased to 84.62% at Visit 2, 38.46% at Visit 3, and zero by Visit 4. Similarly, in the Marketed reference preparation group, at visit 1, 100% of patients used analgesic rescue medication. By visit 2, this decreased slightly to 92.31%, followed by a reduction to 61.54% at visit 3, and 15.38% by visit 4. Initially, 100% of patients in the Placebo group required analgesic rescue medication. This number decreased to 92.31% at visit 2, 65.38% at visit 3, and 38.46% by visit 4.

**Figure 4:**
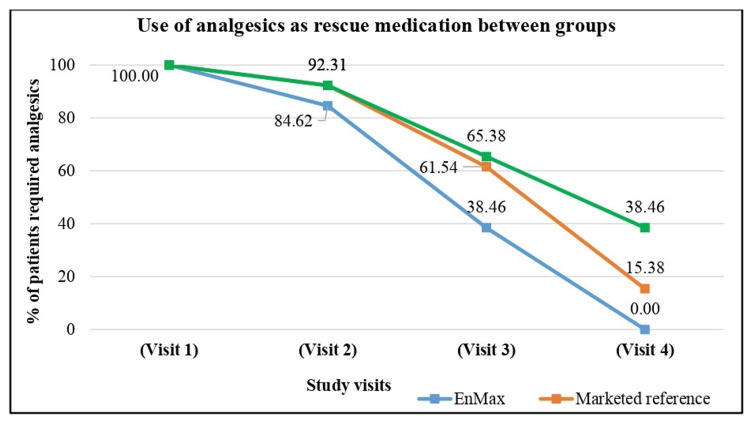
Assessment of changes use of analgesic medications between groups

These results highlight that the EnMax group experienced the most substantial reduction in the use of analgesic rescue medication, reaching zero by visit 4. The Marketed reference group also showed a notable reduction, while the Placebo group exhibited the least decrease in analgesic use over the course of the study despite in Figure 4.

Assessment of symptom grading on a 4-point scale

The assessment of symptoms on a 4-point scale (0 = absent, 1 = mild, 2 = moderate, 3 = severe) revealed notable differences among the EnMax, Marketed reference, and Placebo groups across various parameters (Table 6).

**Table 6:** Assessment of changes in number of patient’s symptom grading on 4-point scale between groups The data was analyzed by using the Chi-square test. Significant at <0.05. * - Denotes a significant value in the comparison between the EnMax and Placebo groups. # - Denotes a significant value in the comparison between the EnMax and Marketed reference groups.

Changes in symptom grading on 4-point scale (number of patient’s)																	
Visits	Visit 1				Visit 2				Visit 3					Visit 4			
Score	0	1	2	3	0	1	2	3	0	1	2	3	0		1	2	3
Symptoms	EnMax																
Erythema	8	8	10	0	13	7	6	0	20^*^	6	0	0	25^*^		1	0	0
Local irritation	4	14	8	0	10	16	0	0	22^*#^	4	0	0	24^*#^		2	0	0
Discharge (Pus/blood)	13	12	1	0	24^*#^	2	0	0	23^*#^	3	0	0	26^*^		0	0	0
Induration	6	11	9	0	12	13	1	0	18^*^	8	0	0	24^*^		2	0	0
Tenderness	4	15	7	0	17^#^	9	0	0	24^*#^	2	0	0	25^*^		1	0	0
Placebo																	
Erythema	9	13	4	0	9	15	2	0	11	15	0	0	12		14	0	0
Local irritation	2	17	7	0	4	18	4	0	7	18	1	0	10		16	0	0
Discharge (Pus/blood)	11	14	1	0	13	13	0	0	15	11	0	0	18		8	0	0
Induration	5	15	6	0	6	18	2	0	10	16	0	0	14		12	0	0
Tenderness	7	13	6	0	10	15	1	0	11	15	0	0	13		13	0	0
Marketed reference																	
Erythema	2	8	3	0	4	8	1	0	7	6	0	0	11		2	0	0
Local irritation	1	9	3	0	3	10	0	0	7	6	0	0	8		5	0	0
Discharge (Pus/blood)	7	5	1	0	8	5	0	0	8	5	0	0	12		1	0	0
Induration	5	6	2	0	6	5	2	0	7	6	0	0	9		4	0	0
Tenderness	2	11	0	0	3	10	0	0	6	7	0	0	10		3	0	0

Erythema, indicative of skin redness, showed significant differences between EnMax and Placebo groups at multiple visits, with EnMax demonstrating faster improvement starting from visit 2 compared to both Placebo and Marketed reference groups. Local irritation also exhibited significant improvements in the EnMax group compared to Placebo by visit 3, with noticeable reductions in symptoms persisting through visit 4. Discharge (pus/blood) levels were significantly lower in the EnMax group from visit 2 onwards compared to both Marketed reference and Placebo groups. Induration and tenderness showed marked reductions in severity in the EnMax group, particularly evident by visit 3, whereas the differences with Marketed reference and Placebo groups were less pronounced.

Overall, EnMax consistently demonstrated superior symptom improvement compared to Placebo, starting as early as visit 2 for some symptoms. While differences with the Marketed reference group were generally less distinct, EnMax consistently showed a trend toward faster and more substantial symptom alleviation across multiple parameters.

Global assessment of response to therapy score after treatment between groups

Patient Response to Therapy

**Figure 5:**
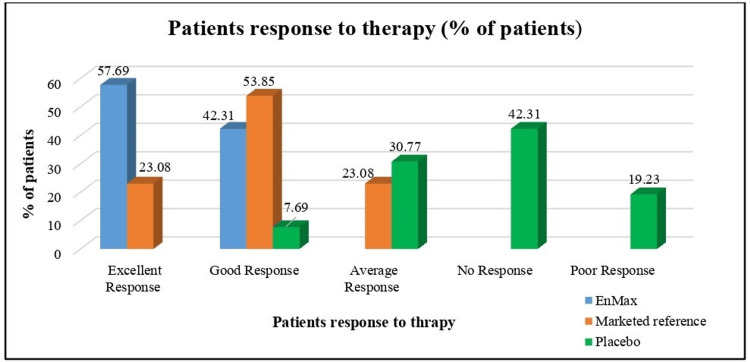
Global assessment of patient response to therapy after treatment between groups

After treatment, patient response to therapy varied significantly across the groups as assessed by the Global Assessment of Patient Response to Therapy scores. In the EnMax group, 57.69% of patients showed an excellent response (score 1), while 42.31% had a good response (score 2). Conversely, in the Marketed reference group, 23.08% demonstrated an excellent response and 53.85% a good response, with fewer patients in the Placebo group showing positive responses of 7.69% and 30.77% for good and average responses, respectively. No (score 4), and poor (score 5) responses were more prevalent in the Placebo group compared to the treated groups (Figure 5).

Investigators' Response to Therapy

**Figure 6:**
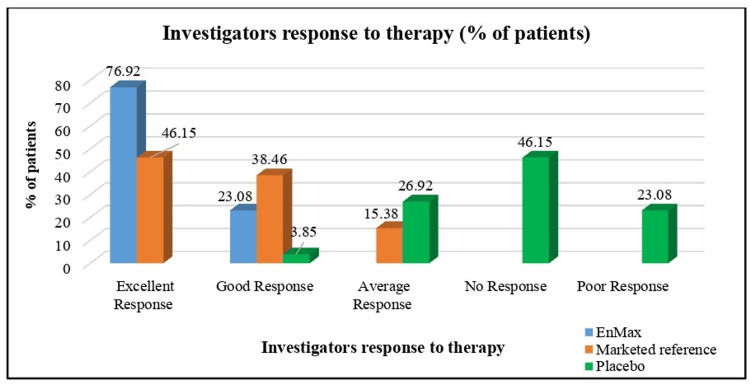
Global assessment of investigators response to therapy after treatment between groups

Similarly, investigator assessments of therapy response (Global Assessment of Investigator Response to Therapy scores) revealed notable distinctions among the groups post-treatment. The EnMax group had higher proportions of excellent (1) and good (2) responses compared to the Marketed reference and Placebo groups. Specifically, 20 patients in the EnMax group received an excellent response rating, followed by six for the Marketed reference group and none for the Placebo group. The Placebo group, in contrast, had a higher number of patients categorized with average (three), no (four), and poor (five) responses, indicating a less favorable outcome compared to the treated groups (Figure 6).

Assessment of complete blood count parameters

All CBC parameters remained well within normal physiological ranges throughout the study. There were no clinically significant changes observed in any of the three groups (EnMax, Marketed, and Placebo). However, it is noteworthy that leukocyte levels were slightly elevated at visit 2 across all groups, likely reflecting an inflammatory or immune response. By visit 4, these levels had returned to within normal ranges.

Assessment of vital signs and compliance

Throughout the study, there were no clinically significant changes observed in vital signs, including blood pressure, heart rate, body temperature, and respiratory rate. All measured parameters remained within normal ranges.

All three groups demonstrated 100% compliance in consuming the investigational product throughout the study and no adverse events were reported during the study.

## Discussion

The randomized, double-blind clinical trial assessed the efficacy of EnMax, a microbial and plant proteolytic enzyme-rutin combination, in managing inflammation and pain in patients undergoing clean surgical procedures. The study included three groups: EnMax, a Marketed reference comparator of trypsin-bromelain-rutoside, and a Placebo, with 65 patients randomized (EnMax: n=26, Marketed reference: n=13, Placebo: n=26). Both male and female patients undergoing elective general, dental, and orthopedic surgeries were included.

EnMax demonstrated the highest statistically significant reduction in inflammation scores across visits (Visit 2: 55.32%, Visit 3: 70.21%, Visit 4: 91.49%). The Marketed reference group followed with reductions of 60%, 68%, and 80%, respectively, while the Placebo group showed reductions of (47.73%, 52.27%, and 63.64%). Although EnMax and the Marketed reference group showed similar effectiveness in inflammation reduction, EnMax was significantly more effective than Placebo by Visit 4, indicating a trend toward efficacy over time.

In terms of pain reduction measured by VRS scores, EnMax exhibited the highest reductions (Visit 2: 72.94%, Visit 3: 81.18%, Visit 4: 95.29%), followed by the Marketed reference group (64.20%, 81.48%, 86.42%) and then the Placebo group (42.42%, 46.06%, 76.97%).

The EnMax group exhibited significant reductions in CRP (51.24%) and ESR (59.80%) levels from Visit 1 to Visit 4, whereas the Marketed reference group showed reductions of 35.23% in CRP and 46.38% in ESR. Both reductions were statistically significant within groups. Between-group analysis revealed EnMax's superiority in CRP reduction compared to both Marketed reference and Placebo groups. These data show EnMax's activity in the reduction of inflammatory biomarkers.

EnMax consistently demonstrated statistically significant improvements in clinical symptoms such as erythema, local irritation, discharge, induration, and tenderness compared to Placebo, beginning as early as Visit 2. Patient and investigator global assessments indicated the perceived superiority of EnMax over the Marketed reference formulation in terms of improvement. No adverse events were reported during the study, indicating the safety and tolerability of EnMax. Compliance with the investigational product was nearly 100% across all groups. There were no clinically or statistically significant changes in hematological and vital signs parameters.

Post-operative pain and inflammation are common after surgical procedures, leading to edema due to increased microvascular permeability and vasodilation. These symptoms are triggered by the body's inflammatory response and can delay healing if uncontrolled [11, 12]. Protease inhibitors alpha1 antitrypsin and alpha2 macroglobulin can inhibit plasmin, preventing fibrinolysis and preventing healing for the competitive inhibition of these Oral combinations of protease enzymes are useful [13]. Controlling swelling is crucial for wound healing and preventing complications. Steroids and NSAIDs are commonly used to manage pain and swelling, but their anti-proliferative effects and limited efficacy limit their usefulness in promoting faster wound recovery [11, 12].

A prospective study of 133 colorectal surgery patients found a 21.8% rate of surgical site infections, with increased risk significantly associated with age over 70, body mass index greater than or equal to 30 kilograms per square meter, American Society of Anesthesiologists scores greater than 2, diabetes, chronic steroid use, and the presence of contaminated or dirty wounds [14].

The study found that the EnMax group experienced a significant reduction in pain and inflammation after surgical corrections, with 77% of patients showing complete resolution of inflammation by day 8. The EnMax group also experienced a reduction in pain score, with over 95% reduction in the EnMax group and 86% reduction in the marketed reference group. This improvement was consistent with global assessments of improvement reported by patients and investigators.

A prospective study of 402 patients undergoing colorectal surgery found that low butyrylcholinesterase levels on the first and third postoperative days were significantly associated with an increased risk of surgical site infections. These results highlight the potential of butyrylcholinesterase as a predictive marker for infections in colorectal surgery [15].

The combination of CRP, ESR, and white blood cell (WBC) counts is useful in predicting the absence of infection after elective surgery [16]. The reduction in CRP levels following hip or knee arthroplasties, a marker of surgical trauma, was observed in a study of total hip replacement (THR) after femoral neck fracture. The oral enzyme combination group experienced a 32% reduction in CRP levels compared to the placebo group, suggesting reduced trauma-induced inflammation and faster recovery [17]. In a clinical trial at Grant Medical College in Mumbai, trypsin was found to be more effective than serratiopeptidase and a combination of trypsin, bromelain, and rutoside in reducing inflammation and promoting wound healing after orthopedic surgery. Trypsin significantly lowered erythema and pain, with 88% of patients rating their response as good to excellent [18]. The EnMax group experienced a 51.24% reduction, while the marketed reference group showed a 35.23% reduction. The reduction in inflammation markers could translate into improved clinical outcomes, such as improved range of movement.

EnMax combines systemic enzymes from microbial and plant proteases with the herbal component Rutin to combat inflammation through multiple mechanisms. Microbial proteases bind to alpha-2-macroglobulin, masking its antigenicity while retaining enzymatic activity, and hydrolyze bradykinin, histamine, and serotonin. Its anti-inflammatory effects are mediated by the regulation of cyclooxygenase 2 and prostaglandin D2, while also inhibiting phospholipase A2 to reduce inflammatory mediators. This results in decreased erythrocyte sedimentation rate, white blood cell, and platelet counts, alongside improved red blood cell count and function.

This study establishes the efficacy of EnMax, a proteolytic enzyme-rutin combination, in managing post-surgical inflammation and pain. EnMax demonstrated similar outcomes compared to both the marketed reference trypsin-bromelain-rutoside combination and superior placebo in reducing inflammation, pain, and inflammatory biomarkers such as CRP and ESR. These findings highlight EnMax’s potential as a highly effective intervention for post-operative inflammation management.

However, limitations include a relatively small sample size, particularly in the Marketed group, which may restrict the generalizability of findings. Variations in surgical techniques and patient characteristics across different sites could introduce variability in outcomes. Given the promising results, further large-scale studies with extended follow-up periods and larger sample sizes are warranted to validate EnMax’s efficacy and safety across various surgical procedures. Additionally, research exploring the application of EnMax in other inflammatory conditions could extend its therapeutic benefits, providing a broader scope for clinical use. The demonstrated safety profile and significant clinical improvements position EnMax as a viable and superior alternative to current post-surgical inflammation management treatments.

## Conclusions

The double-blind, randomized clinical trial demonstrated that EnMax significantly outperformed both the marketed reference preparation and placebo in improving post-operative wound healing. EnMax led to the most substantial reduction in inflammation, pain scores, and inflammatory biomarkers (CRP and ESR) by visit 4, with 84.62% of patients experiencing no inflammation and a 95.29% decrease in pain score. Additionally, EnMax showed the highest reduction in the use of analgesics, with no patients needing them by the study's end. Both patients and investigators rated EnMax’s effectiveness as superior, showing excellent response to therapy compared to the marketed reference preparation and placebo.

Overall, EnMax proved to be a highly effective intervention for post-operative wound healing, achieving better outcomes across various parameters compared to the marketed reference preparation and placebo. The marketed reference preparation also showed efficacy, albeit to a lesser degree than EnMax, while the placebo group displayed the least improvement. The study confirmed the safety of EnMax, with no adverse effects reported, making it a promising option for enhancing post-operative recovery.

## References

[1] Demidova-Rice TN, Hamblin MR, Herman IM (2012). Acute and impaired wound healing: pathophysiology and current methods for drug delivery, part 1: normal and chronic wounds: biology, causes, and approaches to care. Adv Skin Wound Care.

[2] Rahman MS, Hasan K, Ul Banna H, Raza AM, Habibullah T (2019). A study on initial outcome of selective non-operative management in penetrating abdominal injury in a tertiary care hospital in Bangladesh. Turk J Surg.

[3] Mohan N, Gnanasekar D, Tk S, Ignatious A (2023). Prevalence and risk factors of surgical site infections in a teaching medical college in the Trichy district of India. Cureus.

[4] Zabaglo M, Sharman T (2024 Jan-). Postoperative wound infection. StatPearls [Internet].

[5] Culver DH, Horan TC, Gaynes RP (1991). Surgical wound infection rates by wound class, operative procedure, and patient risk index. National Nosocomial Infections Surveillance System. Am J Med.

[6] Monika P, Waiker PV, Chandraprabha MN, Rangarajan A, Murthy KN (2021). Myofibroblast progeny in wound biology and wound healing studies. Wound Repair Regen.

[7] McCarty SM, Percival SL (2013). Proteases and delayed wound healing. Adv Wound Care (New Rochelle).

[8] Nauroy P, Nyström A (2020). Kallikreins: Essential epidermal messengers for regulation of the skin microenvironment during homeostasis, repair and disease. Matrix Biol Plus.

[9] Latha B, Ramakrishnan KM, Jayaraman V (1997). Action of trypsin: chymotrypsin (Chymoral forte DS) preparation on acute-phase proteins following burn injury in humans. Burns.

[10] Akhtar NM, Naseer R, Farooqi AZ, Aziz W, Nazir M (2004). Oral enzyme combination versus diclofenac in the treatment of osteoarthritis of the knee--a double-blind prospective randomized study. Clin Rheumatol.

[11] Sisodia Y, Dharangutti R, Khemnar B Post-operative management of inflammation after orthopaedic surgeries using trypsin, bromelain and rutoside combination: a single-centre prospective observational study. Int J Res Orthop.

[12] Tiwari. S, Khemnar. BM, John. J (2022). Management of post-operative wound in dental surgeries using proteolytic enzyme-flavonoid combination of trypsin, bromelain and rutoside: a single-centre experience. Int J Res Med Sci.

[13] Chakraborty S, Roy P (2019). Peri-operative role of proteases (bromelain + rutosides) in surgical patients- a prospective clinical trial. Asian J Sci Tech.

[14] Panos G, Mulita F, Akinosoglou K (2021). Risk of surgical site infections after colorectal surgery and the most frequent pathogens isolated: a prospective single-centre observational study. Med Glas (Zenica).

[15] Verras GI, Mulita F (2024). Butyrylcholinesterase levels correlate with surgical site infection risk and severity after colorectal surgery: a prospective single-center study. Front Surg.

[16] Kunakornsawat S, Tungsiripat R, Putthiwara D, Piyakulkaew C, Pluemvitayaporn T, Pruttikul P, Kittithamvongs P (2017). Postoperative kinetics of C-reactive protein and erythrocyte sediment rate in one-, two-, and multilevel posterior spinal decompressions and instrumentations. Global Spine J.

[17] Youn G, Choi MK, Kim SB (2022). Comparison of inflammatory markers changes in patients who used postoperative prophylactic antibiotics within 24 hours after spine surgery and 5 days after spine surgery. J Korean Neurosurg Soc.

[18] Chandanwale A, Langade D, Sonawane D, Gavai P (2017). A randomized, clinical trial to evaluate efficacy and tolerability of trypsin:chymotrypsin as compared to serratiopeptidase and trypsin:bromelain:rutoside in wound management. Adv Ther.

